# The Cdc42 effectors Gic1 and Gic2 regulate polarized post-Golgi secretion

**DOI:** 10.1186/s13578-019-0295-x

**Published:** 2019-04-04

**Authors:** Ying Liu, Tianrui Zhang, Dong Sun, Guangzuo Luo

**Affiliations:** 1 Department of Biochemistry and Molecular Biology, China Medical University, Shenyang, 110122 China; 2 Institute of Translational Medicine, China Medical University, Shenyang, 110122 China

**Keywords:** Cdc42, Gic proteins, Sec3, Exocytosis, Exocyst

## Abstract

**Background:**

Cell polarity refers to spatial difference in morphology, structure, and function within different parts of a single cell, which plays important roles in a wide range of cellular processes. In eukaryotic cells, the small GTPase Cdc42 and phosphatidylinositol 4,5-bisphosphate (PtdIns(4,5)P_2_) are critical components for cell polarity and required for polarized exocytosis and cell growth. Previous data showed that the GTPase-interacting components, Gic1 and Gic2, control cell polarity through its binding with Cdc42 and PtdIns(4,5)P_2_ in the plasma membrane in budding yeast. However, whether the Gic proteins regulate polarized exocytosis is unknown.

**Results:**

In this study, we found that Gic2 co-immunoprecipitates with the exocyst complex, suggesting Gic proteins may be involved in exocytosis. Although we could not show the direct interaction between Gic2 and exocyst, we found *gic1Δgic2Δ* are synthetically sick with *sec3ΔN*. We demonstrated that Gic1 and Gic2 are required for polarized exocytosis in a yeast strain harboring the N-terminal domain deletion of Sec3, which is also known as an effector of Cdc42 GTPase. Gic proteins are required for polarized localization of exocyst, growth, and efficient secretion in *sec3∆N* mutant. In addition, we found that the N-terminal domain of both Gic2 and Sec3 share the similar binding sites of Cdc42. Surprisingly, not all the Sec3/Gic binding deficient *cdc42* mutants displayed defects of growth and secretion, indicating that disruption of Cdc42 binding with Gic proteins and Sec3 does not necessarily show secretion defects in *cdc42* mutants.

**Conclusions:**

We conclude that Gic1/2 and Sec3 act in parallel to regulate polarized post-Golgi secretion, but this regulation is not solely controlled by their upstream factor Cdc42. Considering that N-terminal domain of Gic2 and Sec3 can bind to both Cdc42 and PtdIns(4,5)P_2_, the regulation of Gic protein and Sec3 on polarized secretion may also be controlled by PtdIns(4,5)P_2_. Further experiments need to be performed to test this hypothesis. Our findings provide important clues for understanding the molecular mechanism of cell polarity establishment in eukaryotic cells.

**Electronic supplementary material:**

The online version of this article (10.1186/s13578-019-0295-x) contains supplementary material, which is available to authorized users.

## Introduction

Cell polarity is a common feature for most if not all eukaryotes. It is essential for many processes such as cell growth, differentiation, and morphogenesis, which rely on successful implementation of polarized secretion [1–4]. Previous studies indicated that Cdc42 controls polarized post-Golgi secretion [5–7]. A key downstream effector of Cdc42 is the exocyst [7–10], which is an evolutionarily conserved octameric protein complex that mediates the tethering of post-Golgi secretory vesicles to the plasma membrane prior to fusion [11–14]. The exocyst subunits are localized to sites of active secretion and cell surface expansion. In the budding yeast, the exocyst is localized to the bud tip during early stages of daughter cell growth, and the mother–daughter cell junction (“neck”) during cytokinesis. The exocyst subunits Sec3 and Exo70 directly interact with PtdIns(4,5)P_2_ and the Rho family of GTPases at the plasma membrane [9, 10, 15–21]. The assembly of the exocyst complex mediates the tethering of the secretory vesicles to the plasma membrane for exocytosis [22–25]. Cdc42, in its GTP-bound state, interacts with the N-terminus of Sec3 [7, 8]. Disrupting the Sec3–Cdc42 interaction leads to synthetic defects in secretion in a background where the cells are mutated in another exocyst subunit, Exo70, which was also shown to interact with Cdc42 [9, 10, 17, 18].

The GTPase-interacting components Gic1 and Gic2, also called Gic proteins, share 39% identity and 54% similarity in protein sequence, and play critical role for establishment of cell polarity. They are involved in initiation of budding and cellular polarization through interacting with Cdc42 via the Cdc42/Rac-interactive binding (CRIB) domain and with PI(4,5)P_2_ via a polybasic region [10, 26–30]. Although it has been shown that the lethality of the *sec3∆N exo70*-*38* double mutant can be rescued by adding the N terminus of Gic2 to *sec3∆N* [10], it is not clear whether Gic proteins is involved in polarized secretion.

In this study, we find that Gic2 physically interacts with exocyst complex, and Gic proteins are required for polarized exocytosis in a mutant strain harboring the deletion of Sec3N-terminal domain. But the disruption of Cdc42 binding with Gic proteins and Sec3 does not necessarily show secretion defects and growth inhibition in *cdc42* mutants. Our data suggests that Gic proteins may regulate polarized secretion by the coordination of both Cdc42 and PtdIns(4,5)P_2_.

## Results

### Gic2 co-immunoprecipitates with the exocyst complex

**Fig. 1:**
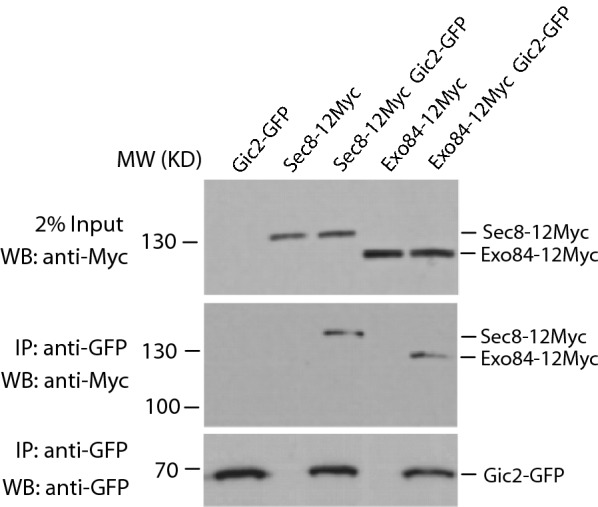
The exocyst complex co-immunoprecipitates with Gic2. Yeast strains expressing Sec8-myc or Exo84-myc were transformed with GFP-tagged Gic2. Cells were immunoprecipitated with polyclonal anti-GFP antibody, and probed with anti-myc monoclonal antibody or anti-GFP monoclonal antibody in western blot. Top panel shows the full-length Sec8-myc and Exo84-myc in the lysates. The middle panel shows that Sec8-myc and Exo84-myc were co-immunoprecipitated in cells expressing GFP-tagged Gic2, but not in cells expressing un-tagged Gic2. The bottom panel shows the Gic2-GFP immunoprecipitated by the anti-GFP polyclonal antibody. GFP-tagged Gic2 and cells expressing un-tagged Sec8 or Exo84 were used as negative control. The representative results from three independent experiments are shown

Because both Sec3 and Gic proteins bind to Cdc42 via the Cdc42/Rac-interactive binding (CRIB) domain and with PI(4,5)P_2_ via a polybasic region [10, 26, 27, 29, 30], and lethality of the *sec3∆N exo70*-*38* double mutant can be rescued by adding the N terminus of Gic2 to *sec3∆N* [10], we speculate that Gic protein may be involved in targeting the exocyst complex to the polarized sites as Sec3N-terminal domain does. To test this hypothesis, we first examined whether Gic1 and/or Gic2 physically associate exocyst in the cells. GFP-tagged Gic2 was expressed in wild type yeast strain, in which the endogenous Sec8 or Exo84 was tagged with the myc epitope by chromosomal integration. Immunoprecipitation was performed using an anti-GFP antibody to detect the association of Gic2 with the exocyst complex. As shown in Fig. 1, the exocyst complex, as represented by the myc-tagged Sec8 and Exo84, co-immunoprecipitated with Gic2-GFP. This result suggests that Gic proteins are physically associated with exocyst complex and may be involved in exocytosis.

### Gic proteins are required for polarized localization of exocyst in *sec3∆N* mutant

**Fig. 2:**
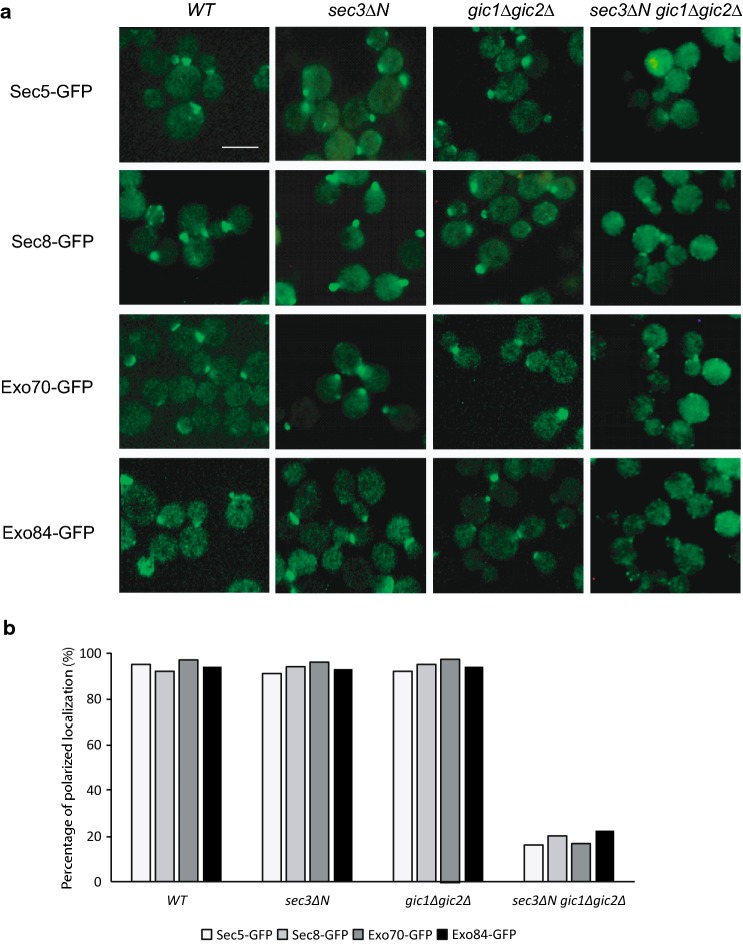
Localization of the exocyst components in the *sec3∆N gic1∆ gic2∆* triple mutant. **a** Polarity of exocyst components are disrupted in *sec3∆N gic1∆ gic2∆* triple mutant, but not in *sec3∆N* single mutant and *gic1∆ gic2∆* double mutant. The exocyst components in wild type, *sec3∆N*, *gic1∆gic2∆*, and *sec3∆N gic1∆ gic2∆* mutant cells were GFP tagged by chromosomal integration. Cells were grown to log phase at 25 °C and then fixed for fluorescence microscopy. Scale bar, 5 μm. GFP-tagged Sec5, 8, and 70 and Exo84 remained polarized to the bud tip in wild type, *sec3∆N*, *gic1∆gic2∆*, but were no longer polarized in *sec3∆N gic1∆ gic2∆* triple mutants. **b** Quantification of the percentage of cells with polarized exocyst subunits in (**a**). 100 cells were counted per group. The representative results from three independent experiments are shown

It was reported that Gic1 and Gic2 are localized specifically to the bud tip, and their localization requires Cdc42 and PtdIns(4,5)P_2_ binding, but is mostly independent of actin [26–28, 30]. Since Gic proteins control cell polarity and Gic2 physically interacts with exocyst complex, we wonder whether Gic1 and Gic2 regulate polarized localization of exocyst as Sec3N-terminal domain does. The *sec3∆N* mutant protein was not able to bind to Cdc42 and PI(4,5)P_2_ in vitro, but the exocyst subunits were polarized to bud tip in this mutant strain [10]. We examined the localization of C-terminal green fluorescent protein (GFP) tagged exocyst subunits, Sec5, Sec8, Exo70 and Exo84, in the strains of wild type, *sec3∆N*, *gic1∆ gic2∆* and *gic1∆ gic2∆ sec3∆N*. As shown in Fig. 2, the exocyst subunits were polarized to bud tip in *gic1∆ gic2∆* and *sec3∆N*, which is similar to that in wild type cells. In *gic1∆ gic2∆ sec3∆N* triple mutant cells, however, most exocyst proteins were either depolarized or diffused inside the cells. We also examined the actin pattern in these mutant strains, and found that actin organization is mostly polarized in these mutants at 25 °C (Additional file 1: Figure S1). In addition, the assembly of the exocyst complex was unaffected in *sec3∆N*, *gic1∆gic2∆*, and *sec3∆N gic1∆gic2∆* triple mutant (Additional file 2: Figure S2). These data suggest that Gic2 and its a paralog, Gic1, may function in parallel with Sec3 for exocyst targeting to the polarized growth sites.

### Deletion of the Gic proteins causes growth and secretion defects in *sec3∆N* mutant

**Fig. 3:**
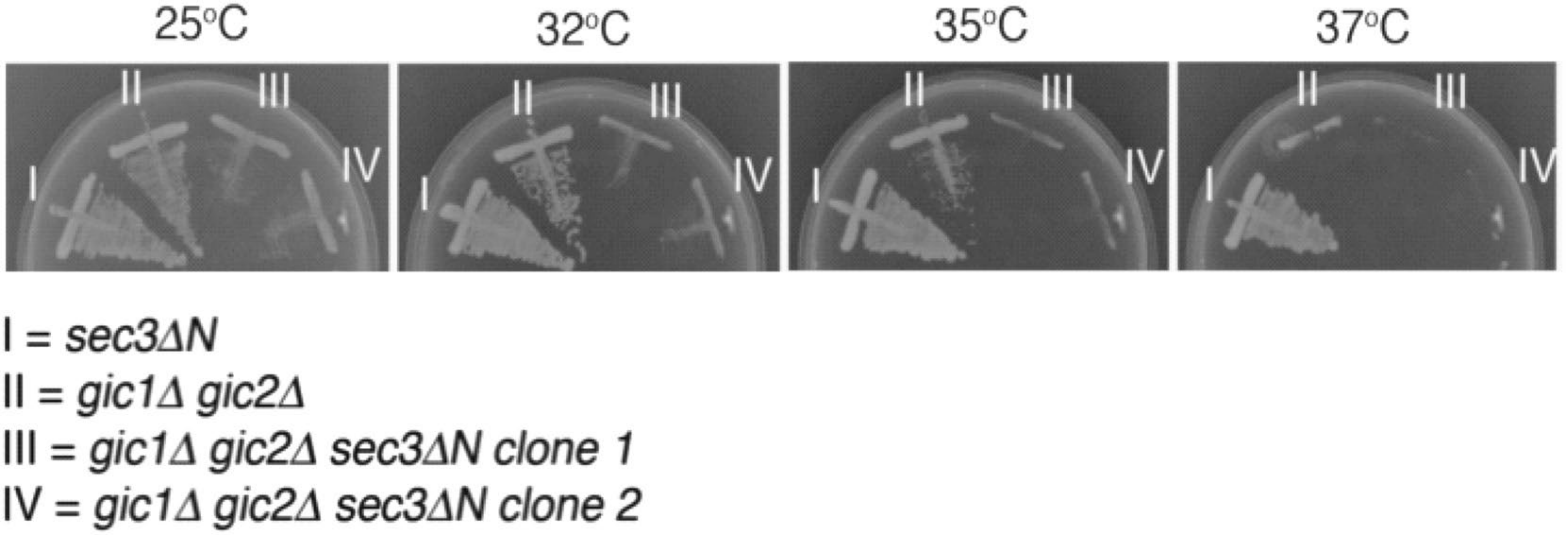
The *gic1∆ gic2∆* double mutant has a synthetic growth defect with the *sec3∆N* mutant. The *gic1∆ gic2∆* double mutants were transformed with a SEC3 balancer (CEN, URA), SEC3 was deleted from the genome, and then the sec3∆N mutant was transformed into *gic1∆gic2∆sec3∆ SEC3* cells. The transformants were streaked onto 5-FOA plates to select for the loss of the wild type SEC3 balancer. The resulting strains were streaked out onto YPD plates with *sec3∆N* and *gic1∆gic2∆* and incubated for 3 days to test the synthetic effect. Two independent clones (“clone 1” and “clone 2”) were tested. The *sec3∆N gic1∆ gic2∆* strains show severe growth defect at 25 °C, and a loss of viability at elevated temperatures. As controls, *sec3∆N* is normal in growth at all the temperatures tested; the *gic1∆gic2∆* mutant has normal growth at 25 °C, has slower growth at higher temperatures, and cannot survive at 37 °C. The representative results from three independent experiments are shown

Because Gic proteins are required for polarized localization of exocyst in *sec3∆N* mutant, we speculate that Gic2 and its a paralog, Gic1, may function in parallel with Sec3 for post-Golgi secretion, and a strain with deletion of Gic1 and Gic2 would have synthetic growth effect with *sec3∆N* mutant. To test this hypothesis, we deleted *GIC1* and *GIC2* either alone or in a *sec3∆N* mutant background yeast strain. As shown in Fig. 3, this combination resulted in a more severe growth defect than either *gic1∆ gic2∆* or *sec3∆N* alone at 25 °C, 32 °C, 35 °C and 37 °C. This result suggests that Gic1 and Gic2 may be involved in regulating exocyst complex in a pathway parallel to Sec3.

**Fig. 4:**
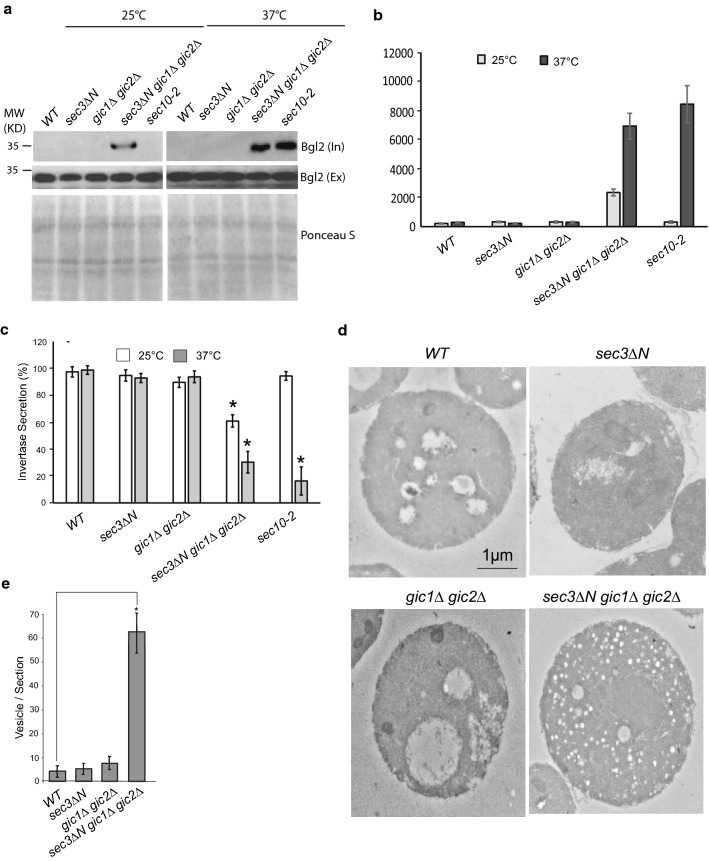
Deletion of the Gic1 and Gic2 in *sec3* mutant cells defects in exocytosis. **a** *sec3∆Ngic1∆gic2∆* triple mutant cells have Bgl2 secretion defects at 25 °C, with a significantly greater defect at 37 °C. Single, double, and triple mutants, as well as *sec10*-2 mutants that are known to have Bgl2 secretion defects at 37 °C, were grown in YPD at 25 °C to early log phase and then grown at 25 °C or shifted to 37 °C for 90 min. The internal and external pools of Bgl2 are detected by Western blot using anti-Bgl2 antibody, and total protein on the PVDF membrane stained with Ponceau S was used as a loading control. **b** Quantification of the amounts of Bgl2 accumulation in cytosol in (**a**). Error bars represent standard deviation. n = 3. **c** *sec3∆Ngic1∆gic2∆* triple mutant cells have invertase secretion defects. Single, double, and triple mutants, as well as *sec10*-2 mutants were grown in YPD at 25 °C to early log phase and then grown at 25 °C or shifted to 37 °C for 90 min and the invertase secretion of each strain was measured. *p < 0.01. Error bars represent standard deviation. n = 3. **d** Triple mutants accumulated a large number of vesicles. Single, double and triple mutants were grown in YPD at 25 °C to early log phase. Cells were fixed and imaged using an electron microscope. **e** Quantification of images in (**d**). 10 cells were counted per group, bars represent standard error. *p < 0.05, student’s t-test

To determine whether these mutations cause secretion defects, we have analyzed the secretion of two cargoes, the cell wall endo-glucanase Bgl2 and the periplasmic enzyme invertase, which are well-characterized markers for the two major classes of post-Golgi vesicles that deliver proteins to the cell surface [31]. As shown in Fig. 4a, Bgl2 accumulation in cytosol (internal) was minimal in the *sec3∆N* and *gic1Δ gic2Δ* double mutant at 25 °C, and a small amount of Bgl2 was accumulated in the *sec3∆N gic1Δ gic2Δ* triple mutant. After shifting to 37 °C for 2 h, although the *sec3∆N* and *gic1Δ gic2Δ* double mutants still did not accumulated Bgl2 protein inside cytosol, the *sec3∆N gic1Δ gic2Δ* triple mutant showed a pronounced amount of Bgl2 accumulation. As a control, the *sec10*-*2* cells did not accumulate Bgl2 at 25 °C, but accumulate Bgl2 at 37 °C. These results indicate that combining *sec3∆N* with *gic1Δ gic2Δ* greatly aggravates the Bgl2 secretion defects.

We also examined the secretion of the periplasmic enzyme invertase in the *sec3∆N*, *gic1Δ gic2Δ* double mutant, and *sec3∆N gic1Δ gic2Δ* triple mutant. As shown in Fig. 4b, at both 25 °C and 37 °C, the wild type, *sec3∆N* or *gic1Δ gic2Δ* mutant cells secreted > 85% of the total invertase outside of cytosol. However, only about 62% of invertase in *sec3∆N gic1Δgic2Δ* triple mutant cells was secreted outside at 25 °C. After a 2-h shift to 37 °C, the triple mutant cells only secreted about 30% of the total invertase.

To examine whether exocytic vesicles are accumulated in *sec3∆N*, *gic1Δ gic2Δ* double mutant, and *sec3∆N gic1Δ gic2Δ* triple mutant, we performed thin-section electron microscopy on these mutants. As shown in Fig. 4c, d, at 25 °C, there were less than 10 vesicles accumulated in *sec3∆N* and *gic1Δ gic2Δ* double mutant, whereas the *sec3∆N gic1Δ gic2Δ* triple mutant accumulated about 62.9 ± 9 vesicles/section vesicles. These results clearly show that Sec3 and Gic1/2 function at parallel to regulate exocytosis.

### Interaction between Gic2 and Cdc42 mutants defective in Sec3-binding is reduced

Since Gic proteins and Sec3 are downstream effectors of Cdc42, we want to know whether the regulation of Gic proteins and Sec3 on polarized exocytosis is under the control of Cdc42.

**Fig. 5:**
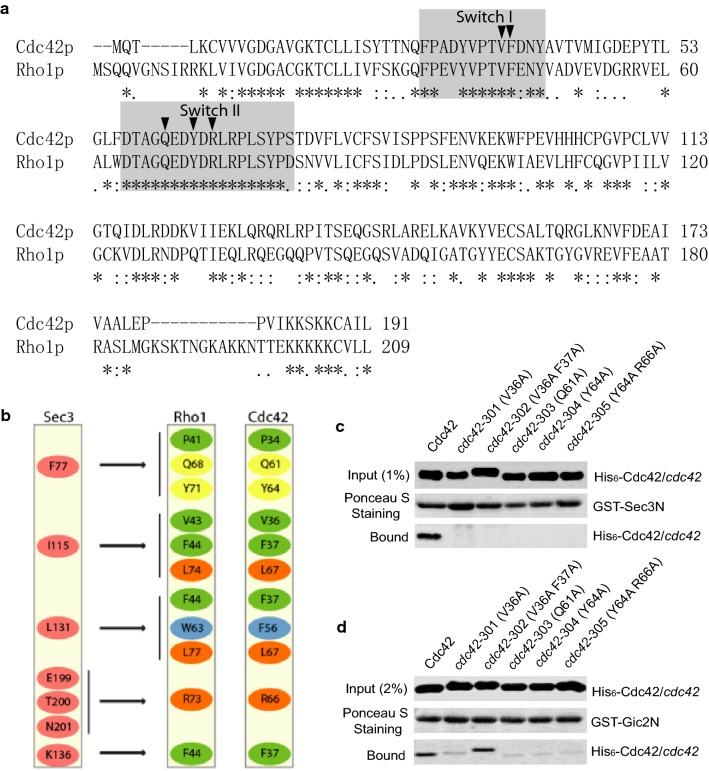
The *cdc42* mutants defect in binding to Sec3N and Gic2N in vitro. **a** Amino acid sequence alignment of the *S. cerevisiae* Cdc42 and Rho1. Identical residues are indicated as asterisks. Switch I and II of Cdc42 and Rho1 are colored gray. Carrots point to amino acids in Cdc42 that have been mutated: positions 36, 37, 61, 64 and 66. **b** Schematic drawing of interactions between Sec3 and Rho1 (adapted from [32]), aligned beside the corresponding amino acid in Cdc42. **c** The *cdc42* mutants were not able to bind to Sec3N in vitro. GST fusion proteins containing (a.a.71–241) of Sec3 was purified and conjugated to glutathione Sepharose. Cdc42, *cdc42*-*301*, *cdc42*-*302*, *cdc42*-*303*, *cdc42*-*304 and cdc42*-*305* were expressed as a Hisx6 fusion and purified from bacteria. The in vitro binding assay was performed using GST-Sec3 and Hisx6-Cdc42 in the presence of GTPγS. The Hisx6-Cdc42 fusion protein bound to the GST-Sec3N Sepharose was detected by Western blotting with anti-Hisx6 antibody (bottom). Equal amounts of Sec3 fusion proteins on beads were used in the binding assay (middle; Ponceau S staining). Equal amounts of cdc42 wild type and mutants were used (top, 1% input, Western blotting with anti-Hisx6 antibody). The representative results from three independent experiments are shown. **d** The *cdc42* mutants defect in binding to Gic2N in vitro. GST fusion proteins containing (a.a.1–155) of Gic2 were purified and conjugated to glutathione Sepharose. Cdc42, *cdc42*-*301*, *cdc42*-*302*, *cdc42*-*303*, *cdc42*-*304* and *cdc42*-*305* were expressed as a Hisx6 fusion and purified from bacteria. The in vitro binding assay was performed using GST-Gic2 and Hisx6-Cdc42 in the presence of GTPγS. The Hisx6-Cdc42 fusion protein bound to the GST-Gic2N Sepharose and was detected by Western blotting with anti-Hisx6 antibody (bottom). Equal amounts of Gic2 fusion proteins on beads were used in the binding assay (middle; Ponceau S staining). Equal amounts of Cdc42 wild type and mutants were used (top, 2% input, Western blotting with anti-Hisx6 antibody). The representative results from three independent experiments are shown

The exocyst subunit Sec3 was proposed to be a landmark on the plasma membrane for exocytosis [6, 15], and is a direct downstream effector of Cdc42 [7, 10]. Sec3 interacts with Cdc42 through a PH domain-like region at its N-terminus [8, 10, 32]. Although some Sec3-binding deficient cdc42 mutants were reported, the binding sites inside Cdc42 and Sec3 is unknown. Besides Cdc42, Sec3 also interacts with Rho1 [16]. Both Cdc42 and Rho1 belong to the Rho family of small GTPases. Rho1 and Cdc42 in *Saccharomyces cerevisiae* share a high degree of sequence similarity. The crystal structure of the N-terminus of Sec3 (“Sec3N”) alone and the Sec3N–Rho1 complex were resolved [8, 32]. The region of Rho1 that interacts with Sec3N is almost identical to a corresponding region of Cdc42 (Fig. 5a), consistent with the previous finding that Cdc42 and Rho1 compete in their binding to Sec3 [7]. Yamashita et al. determined that the mutations in the Switch I and Switch II regions (V43A, F44A, Q68A, Y71A and R73A) in Rho1 would disrupt its binding to Sec3 [32]. Based on the Sec3-binding region of Rho1 (Fig. 5b), we mutated the corresponding amino acids in Cdc42 to alanine by site-directed mutagenesis. Five *cdc42* mutants were made in this study to confirm its binding to Sec3: *cdc42*-*301* (V36A), *cdc42*-*302* (V36A F37A), *cdc42*-*303* (Q61A), *cdc42*-*304* (Y64A) and *cdc42*-*305* (Y64A R66A). Our in vitro binding assay showed that these *cdc42* mutants were defective in binding to GST-Sec3N (a.a.71–241) (Fig. 5c).

The N-terminus of Gic2 contains the classic Cdc42/Rac1 interactive binding (CRIB) domain [26, 27]. Because both Sec3 and Gic1/2 are Cdc42 downstream effectors and lethality of the *sec3∆N exo70*-*38* double mutant can be rescued by adding the N terminus of Gic2 to *sec3∆N* [10], we tested whether any of the *cdc42* mutants defective in Sec3N-binding exhibited altered binding to Gic2. We chose to focus on Gic2 because it was reported that, like Sec3, Gic2 can associate with the plasma membrane through binding to Cdc42 or directly binding to PtdIns(4,5)P_2_, and these associations are important for polarized cell growth [30]. The in vitro binding assay of *cdc42* mutants was performed using GST-tagged Gic2N (amino acids 1–155), which contains its Cdc42-interacting domain [30]. As shown in Fig. 5d, except for *cdc42*-*302*, the binding of GST-Gic2N to the other four *cdc42* mutants decreased dramatically. These results suggest that the binding sites of Cdc42 in Gic2 is very similar to Sec3.

### Sec3-binding deficient Cdc42 mutants displayed different characteristics at growth and secretion

**Fig. 6:**
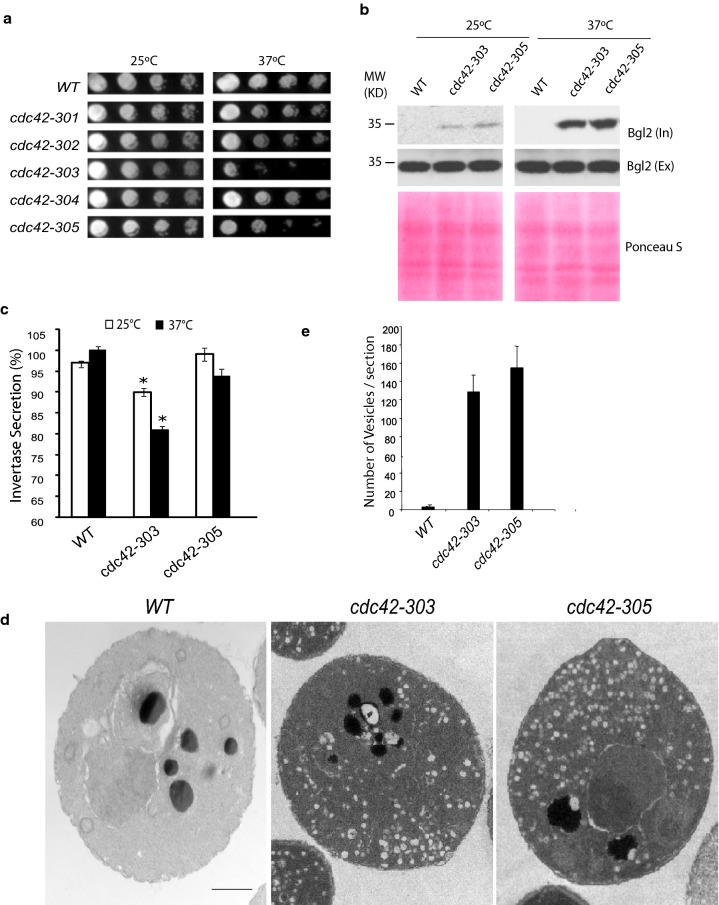
Growth and Secretion defects in *cdc42* mutant alleles. **a** Temperature-dependent growth of *cdc42* mutants. Equal densities of wild-type or *cdc42* mutant cells were spotted in tenfold serial dilutions from left to right on YPD plates and incubated at 25 °C or 37 °C. *cdc42*-*303* and *cdc42*-*305* had severe growth defects at 37 °C. **b** *cdc42*-*303* and *cdc42*-*305* have Bgl2 secretion defects at both 25 °C and 37 °C. Wild-type and *cdc42* mutants are grown in YPD at 25 °C to early log phase and then grown at 25 °C or shifted to 37 °C for 90 min. The internal and external pools of Bgl2 are detected by Western blot using anti-Bgl2p antibody. **c** *cdc42*-*303* has invertase secretion defects at both 25 °C and 37 °C. *p < 0.001 **d** Thin-section transmission EM of wild type, *cdc42*-*303* and *cdc42*-*305*. Cells were grown at 25 °C and shifted at 37 °C for 90 min. **e** Quantification of cells who have large amount of secretion vesicles in GY3670 (*cdc42*-*303*) and GY3672 (*cdc42*-*305*). 10 cells were counted for each group. Error bars represent standard deviations. The representative results from three independent experiments are shown

To evaluate the physiological role of the interaction of Cdc42 with both Sec3 and Gic proteins, we have expressed the Sec3-binding deficient Cdc42 mutants in *Saccharomyces cerevisiae* under control of the endogenous Cdc42 promoter. The growth of the mutant cells was examined on YPD plates at 25 °C or 37 °C (Fig. 6a). The five *cdc42* mutants grew well as wild type cells at 25 °C. At 37 °C, however, *cdc42*-*303* and *cdc42*-*305* mutants show growth defects, while the growth rate of the other three *cdc42* mutants is similar to wild type strain.

To examine whether the growth defect was an outcome of secretion inhibition in *cdc42*-*303* and *cdc42*-*305* mutants, we examined the secretion of the cell wall enzyme Bgl2 and the periplasmic enzyme invertase. The *cdc42*-*303 and cdc42*-*305* cells showed mild defect of Bgl2 secretion at 25 °C, and the secretion in these mutants was seriously inhibited at 37 °C (Fig. 6b). However, only *cdc42*-*303* but not *cdc42*-*305* showed a partial but statistically significant invertase secretion defect at both 25 °C and 37 °C (Fig. 6c). It is not surprising that Cdc42 may regulate only one type of exocytic cargo under certain condition. Adamo et al. [5] observed that the *cdc42*-*6* mutant had a more profound defect in Bgl2 secretion than invertase. Our results are consistent with this observation.

To further confirm the secretion defects in *cdc42*-*303* and *cdc42*-*305* mutants, the two mutants were examined by electron microscopy. Post-Golgi vesicles, which are typically 80–100 nm in diameter, accumulated about 127 and 155 vesicles per section in *cdc42*-*303* and *cdc42*-*305* mutants at 37 °C, respectively (Fig. 6d, e). These results indicate the secretion in *cdc42*-*303* and *cdc42*-*305* mutants was inhibited.

Taken together, the disruption of Cdc42 with Sec3 and Gic1/2 does not necessarily lead to growth and secretion defects, which means other factor, such as PI(4,5)P_2_, may also controls the regulation of Gic proteins on polarized secretion. The different growth and secretion characteristics among *dc42* mutants in this study also suggests that, except for Sec3 and Gic1/2, other Cdc42 effectors may also involved in polarized secretion.

## Discussion

In this study, we have examined the role of the two Cdc42 effectors, Gic1 and Gic2, in polarized secretion. We found that Gic2 are associated with exocyst complex (Fig. 1). Although we could not show the direct interaction between Gic2 and exocyst components under our experimental conditions, we found that *gic1Δgic2Δ* double mutant is synthetically sick with *sec3ΔN* single mutant and regulates cell gowth and exocytic secretion. Gic proteins regulate polarized secretion by targeting the tethering complex to bud tip of yeast cells (Figs. 2, 4). However, although both Gic proteins and Sec3 are the effectors of Cdc42, disrupting the physical interaction of Cdc42 with these effectors does not necessarily show growth and secretion defect (Fig. 6), indicating Cdc42 is not the only upstream regulator of Gic1/2 and Sec3 who control the polarized secretion.

Although Gic1/2 and Sec3 are considered the downstream effectors of Cdc42, they can also function in the upstream of Cdc42. It was reported that Gic1 and Gic2 may stabilize the Cdc42 effector complex at the bud site and regulate initiation of budding [29]. Orlando et al. reported that transportation of Cdc42 to the bud site depends on the exocytic pathway in yeast [33]. Because Sec3 is the landmark of exocytic secretion [15], it may also regulates polarization of Cdc42. According to these data, it is possible that Gic1 and Gic2 may also regulate polarized exocytic secretion through Cdc42.

Zhang et al. [7, 10] found that Sec3N terminal domain can bind to both Cdc42 and PtdIns(4,5)P_2_. The Cdc42-binding domain in Sec3N-terminus is adjacent to the PtdIns(4,5)P_2_ binding region, and the interaction of Sec3N-terminus with both Cdc42 and PtdIns(4,5)P_2_ are important for Sec3 function. Yamashita et al. [32] also found that *sec3* mutants deficient in the binding of either Rho1 or PtdIns(4,5)P_2_ can still be localized to the bud tip even in the presence of Latrunculin B, which disrupts the actin microfilament cytoskeleton, while the *sec3* mutants deficient in the binding of both Rho1 and PtdIns(4,5)P2 are almost completely depolarized in the presence of Latrunculin B. These results suggest that both Rho GTPase and PtdIns(4,5)P2 can act as receptors for the exocyst complex in a complementary fashion. Gic proteins are considered to be targeted to the bud tip and plays an important role in early bud formation in yeast. The GTP-bound Cdc42 interacts with Gic2 through the Cdc42/Rac interactive binding domain located at the N terminus of Gic2 and activates Gic2 during bud emergence [26, 27]. Orlando et al. [30] have identified a polybasic region in Gic2 adjacent to the Cdc42/Rac interactive binding domain, which directly interacts with PtdIns(4,5)P_2_ in the plasma membrane. This physical interaction is also necessary for the polarized localization of Gic2 protein to the bud tip and is important for the function of Gic2 in cell polarity. These facts indicate that PtdIns(4,5)P_2_ and Cdc42 act in concert to regulate polarized localization and function of Gic2 during polarized cell growth in the budding yeast. Based on these previous reports and the results shown in our study, we speculate that either the Cdc42–Sec3/Gic interaction or PtdIns(4,5)P_2_–Sec3/Gic interaction can targeting the exocyst complex to the polarized growth sites, which explains the result that three Sec3-binding deficient *cdc42* mutants did not show growth defect in Fig. 6, though these *cdc42* mutants can not bind to Sec3 and/or Gic2.

Cdc42, the highly conserved small GTPase in eukaryotic organisms, mediates extracellular signals, triggering changes in transcription and in the actin cytoskeleton organization. Dozens of proteins have been found to act downstream of Cdc42, such as members of the PAK, WASP/WAVE, formin, lipid-kinase, IQGAP and NADPH oxidase families [34]. Although the disruption of interaction of Cdc42–Sec3/Gic is not sufficient to trigger secretion inhibition, these Sec3/Gic-binding deficient *cdc42* mutants may also be defective in interaction with other effectors. It is possible that the interaction of Cdc42 with other effectors are also destroyed in the two *cdc42* mutants, *cdc42*-*303* and *cdc42*-*305*. But it is unlikely that the ATP loading capacity of these mutants is the main reason for the binding defect because no interaction was detected between c*dc42* mutants and Sec3, and neither GDP nor GTP affected the binding of Sec3 to all five mutants (Additional file 3: Figure S3). In addition, even if the ATP loading capacity of some *cdc42* mutants is defective, for example *cdc42*-*303 and cdc42*-*305*, this effect on the binding of Cdc42 to Sec3 and Gic1/2 is generic to all the downstream effectors, not specific to Sec3 and Gic1/2.

To exclude the possibility that actin, instead of Gic proteins, are responsible for the secretion defects in *gic1∆gic2∆sec3∆N* triple mutant cells. The actin pattern was also examined in *sec3∆N*, *gic1∆gic2∆* double mutant, and *gic1∆gic2∆sec3∆N* triple mutant. The actin organization in *sec3∆N* cells is polarized as expected (Additional file 1: Figure S1). Although the Gic proteins are required for actin cytoskeletal polarization, the polarized actin pattern is only slightly affected at non-restrictive temperature [26, 27]. Consistent with these results, we did not find apparent secretion defect in *sec3∆N* single mutant and *gic1∆gic2∆* double mutant at 25 °C (Fig. 4). Because the actin pattern in *gic1∆gic2∆sec3∆N* triple mutant is similar to *gic1∆gic2∆* double mutant at 25 °C (Additional file 1: Figure S1), the secretion defect in *gic1∆gic2∆sec3∆N* triple mutant would not be the outcome of disruption of polarized actin pattern.

We found that Gic2 is physically associated with exocyst complex. It is likely that its paralog, Gic1, may also interacts with this protein complex. However, it is unclear whether Gic proteins bind directly to one of the exocyst subunits, or bind to another protein which interacts with exocyst complex. Another exocyst subunit, Exo70, binds to both Cdc42 and PtdIns(4,5)P_2_ and regulates polarized secretion [9, 17, 18, 21]. Because the binding of these two subunits with Cdc42 and PtdIns(4,5)P_2_ are required for targeting of exocyst to bud tip, we wanted to know whether Gic proteins regulate polarized secretion through its binding with Exo70. However, we did not detect any physical interaction of Gic2 with Exo70 both in vivo and in vitro (data not shown). Further experiments are needed to explore the molecular mechanism of Gic proteins interacting with exocyst complex.

## Experimental procedures

### Plasmids and yeast strains

Standard methods were used for yeast growth and genetic manipulations [35]. All strains used in this project are listed in Additional file 4: Table S1. Yeast transformation was performed based on the lithium acetate method [36]. 2.5 A_600_ units of early log phase cells were collected in a 1.5-ml centrifuge tube and washed once with 0.5 ml of distilled water. 240 μl PEG 3350 (50% wt/vol), 36 μl 1.0 M LiAc, 5 μl 10 mg/ml sonicated salmon sperm DNA (Agilent Technologies), and 0.1–10 μg DNA were added and the tube was vortexed until the cell pellet had been completely mixed. The cells were then shocked in a water bath at 42 °C for 20–25 min. 2–200 μl of the transformation mix was plated onto Petri dish plates containing solid synthetic complete medium. The plates were incubated at 25 °C for 2–4 days to recover transformants.

All mutagenesis was performed based on the *pRS314*-*CDC42* plasmid [37] using the QuickChange site-directed mutagenesis kit (Stratagene, Inc.) and verified by sequencing. Different mutations were transformed into YEF1194 [37] and the transformants were later selected on SC-Trp-His plates with 5-FOA to lose the *pRS316*-*HA*-*CDC42* plasmid balancer. The strains used in this study are listed in the strain table. Standard methods were used for yeast media and genetic manipulations.

### Light microscopy

Chromosomal tagging of *SEC5*, *SEC8*, *EXO70* and *EXO84* by Green Fluorescence Protein (GFP) was performed as previously described [38]. Cells were grown to log phase at 25 °C. The cells were then fixed for microscopy as described previously [39]. F-actin was stained with Alexa Fluor 488-conjugated phalloidin (Molecular Probes Corp.). Fluorescence microscopy was conducted with microscope CTR6000 (Leica) equipped with a Plan-Apochromat 100x, 1.40 NA oil immersion objective lens. Images were taken using LAS AF 1.5.1 acquisition software (Leica).

### Electron microscopy (EM)

Cells were grown at 25 °C in YPD media to log phase, and were then processed for thin section EM analysis using a Jeol-1010 transmission electron microscope. Cells for EM were collected by vacuum filtration using a 0.45 µm nitrocellulose membrane and were fixed for 1 h at room temperature in fixation buffer (0.1 M cacodylate, pH 7.4, 3% formaldehyde, 1 mM MgCl_2_, and 1 mM CaCl_2_). The cells were spheroplasted and fixed with 1% glutaraldehyde (in PBS, pH 7.4) at 4 °C overnight. The spheroplasts were washed in 0.1 M cacodylate buffer and were post-fixed twice with ice-cold 0.5% OsO4 and 0.8% potassium for 10 min each. After dehydration and embedding in Spurr’s epoxy resin (Polysciences, Inc.), thin sections were cut and transferred onto 600 mesh uncoated copper grids (Ernest Fullam, Inc.) and were post-stained with uranyl acetate and lead citrate. Cells were observed on a transmission electron microscope (Model 1010; JEOL) at 100,000× magnification.

### Secretion assays

Analyses of the secretion of Bgl2 and invertase were carried out as previously described [40]. 20 ml of yeast cells were grown to early log phase (OD_600_ is 0.6–1.0) at 25 °C. NaN_3_ and NaF were added directly to the culture at a final concentration of 10 mM each. 10 OD_600_ units of cells were collected, washed with cell wash buffer (20 mM Tris–HCl, pH 7.5, 10 mM NaN_3_, and 10 mM NaF). The cells were resuspended in 300 µl of spheroplast solution buffer (50 mM Tris–HCl, pH 7.5, 1.4 M sorbitol, 10 mM NaN_3_, 10 mM NaF, 30 mM 2-Mercaptoethanol, and 0.2 mg/ml Zymolyase) and incubated at 37 °C in a water bath for 30 min. The spheroplasts were pelleted gently by centrifuge at 2000 rpm for 5 min at 4 °C. 100 µl of supernatant was carefully transferred to a new tube and mixed with 20 µl of 6× SDS loading buffer (300 mM Tris–HCl, pH 6.8, 600 mM dithiothreitol, 12% SDS, 0.6% bromophenol blue, and 60% glycerol). This is the external pool. The remaining supernatant was removed and the pellet (spheroplasts) was washed once with 1 ml of spheroplast wash buffer (50 mM Tris–HCl, pH 7.5, 1.4 M sorbitol, 10 mM NaN_3_, and 10 mM NaF) to remove residue external pool. The spheroplasts were dissolved in 300 µl of lysate buffer (20 mM Tris–HCl, pH 7.5, 100 mM NaCl, 2 mM MgCl2, 0.5% Triton X-100, and 1× protease inhibitor cocktail; Roche) by leaving on ice for 10 min. The cell debris was removed after the sample was centrifuged at 13,000 rpm for 5 min at 4 °C. 100 µl of supernatant (lysates) was transferred to a new tube and mixed with 20 µl of 6× SDS loading buffer. This is the internal pool. The internal pool and external pool samples were boiled at 95 °C for 5 min, loaded into 12% SDSPAGE gel. Bgl2 was detected by Western blotting with an anti-Bgl2 rabbit polyclonal antibody (1:2000). For temperature-sensitive mutants, the cells were grown at 25 °C or shifted to 37 °C for 90 min before being processed for the Bgl2 assay.

Invertase secretion was examined as described previously [41]. 20 ml of yeast cells were grown to early log phase (OD_600_ is 0.6–1.0) at 25 °C. 1 OD cells were transferred to a new tube and washed with 1 ml of ice cold 1 mM NaN_3_. The cells were then resuspended in 1 ml YP plus glucose medium (1% Bacto-yeast, 2% Bactopeptone, and 0.1% glucose) and incubated at 25 °C for 1 h to induce invertase expression. After 1 h of incubation, the cells were collected and washed once with 1 ml 10 mM NaN_3_. The cells were resuspended in 1 ml 10 mM NaN_3_ and kept on ice. The external invertase was measured directly on the whole intact cells, whereas the internal invertase was measured after preparation of lysates. 0.5 ml of cells were removed and mixed with 0.5 ml of 2× spheroplast cocktail mix (2.8 M sorbitol, 0.1 M Tris–HCl pH 7.5, 10 mM NaN_3_, 0.4% 2-Mercaptoethanol, and 10 µg/ml Zymolyase-100T). The cells were incubated in water bath at 37 °C for 45 min. The spheroplasts were collected and the supernatant was removed carefully without disturbing the pellet. The spheroplasts were dissolved at 4 °C in 0.5 ml 0.5% Triton X-100. The invertase assay was performed in 13 × 100 mm tubes. 20 µl of sample was placed in the tube and 80 µl of 50 mM NaAc, pH 5.1, was added. Then 25 µl of 0.5 M sucrose was added and the tube was incubated at 37 °C for 30 min. 150 µl of 0.2 M K_2_HPO_4_ was added and the tube was placed on ice to stop the reaction. The sample was boiled for 3 min and put on ice. 1 ml of assay mix was added (0.1 M KPi buffer, pH 7.0, 10 U/ml glucose oxidase, 2.5 µg/ml peroxidase, 150 µg/ml O-dianisidine, and 20 µM *N*-Ethylmaleimide) and the sample was incubated at 37 °C for 30 min. 1 ml of 6 N HCl was added into the tube and the value of A_540_ was measured by the Spectrophotometer (SmartSpec 3000; Bio-Rad Laboratories). For temperature-sensitive mutants, the cells were grown at 25 °C or shifted to 37 °C for 90 min and then were grown in low-glucose medium (0.1% glucose) at the same temperature for 1 h.

### In vitro binding assay

Amino-terminus of Sec3 (a.a.71–241) and Gic2 (a.a.1–155) were expressed as GST fusion proteins. Cdc42 and *cdc42* were expressed as His_6_-tagged fusion protein (His_6_-Cdc42 or His_6_-*cdc42*). 20 μg Sec3 (a.a.71–241) or Gic2 (a.a.1–155) and 10 μg Cdc42 or *cdc42* mutant were used for in vitro binding assay as previously described [10].

### Whole cell extract preparation

Yeast extracts were prepared using glass beads as described previously [42]. Yeast cells were grown to early log phase and collected by rapid centrifugation. The cell pellet was suspended in ice-cold lysis buffer (50 mM Tris–HCl, pH 7.5, 100 mM NaCl, 1 mM EDTA, 1% Triton X-100, 5 mM NaF, 1 mM sodium pyrophosphate, 1 mM dithiothreitol, and 1× protease inhibitor cocktail; Roche). Glass beads (0.4–0.6 mm in diameter) were added into the resuspended cells. Cells were broken by vigorous vortexing at full speed for 8–10 cycles of 30-s vortexing followed by 30 s in an ice-water bath. The beads and cell debris were removed by centrifugation at 10,000*g* at 4 °C, and the supernatant was further clarified by centrifugation at 100,000*g* at 4 °C. The published Hot-SDS protein extraction protocol was used with slight modifications [43]. In brief, 50 A600 units of yeast cells were collected by centrifugation, washed once with water, and then suspended in 1 ml of cold distilled water, followed by the addition of 1 ml of 0.2 M NaOH. Samples were mixed and incubated for 5 min at room temperature. Cells were collected by centrifugation, resuspended in 1 ml of SDS sample buffer (0.06 M Tris–HCl, pH 8.6, 5% glycerol, 1% SDS, and 10 mM 2-Mercaptoethanol) and boiled for 3–5 min. Samples were centrifuged, and protein concentrations were determined using the DC protein assay kit (Bio-Rad Laboratories).

### Immunoprecipitation

For immunoprecipitation of GFP-tagged Sec5, total proteins were extracted by using the glass beads method [42]. A rabbit anti-GFP antibody was used for immunoprecipitation. Cell lysates were used for immunoprecipitation using protein G beads and anti-GFP antibody in immunoprecipitation buffer (50 mM Tris–HCl, pH 7.5, 150 mM NaCl, 1 mM EDTA, 1 mM EGTA, 10% glycerol, and 1× protease inhibitor; Roche) and 1× phosphatase inhibitor I (Roche) at 4 °C for 4 h with rotation. Beads were washed with washing buffer (50 mM Tris–HCl, pH 7.5, 150 mM NaCl, 1 mM EDTA, 1 mM EGTA, and 10% glycerol) three times. The beads were mixed with 30 µl of 1× SDS loading buffer and incubated at 95 °C for 5 min. Protein samples were then cooled on ice for several minutes and separated on 10% SDS-PAGE gel. Western blotting was performed using the anti-myc, anti-Sec8, Sec10 and Sec15 antibody. The Images were further processed using ImageJ software (National Institutes of Health).

## Additional files

**Additional file 1: Figure S1.** Polarization of actin in *gic1∆gic2∆sec3∆N* mutant. Cells were grown to log phase at 25°C, and then fixed. Actin was stained by Alexa Fluor 488-conjugated phalloidin. Scale bar, 5μm. (B) Quantification of cells with polarized actin in each mutant. 90 cells were counted for each group (n=3). Error bars represent standard deviations. The representative results from three independent experiments are shown.

**Additional file 2: Figure S2.** Structural integrity of the exocyst complex in the *gic1∆gic2∆sec3∆N* triple mutant. The exocyst component Sec5 in wild-type and mutant strains was GFP-tagged by chromosomal integration. The cells grown to log phase were incubated at 25°C or shifted to 37°C for 2 h. The exocyst was immuno-isolated from the wild type and mutant cells by anti-GFP antibody, and individual exocyst components were detected by Western blot. At both 25°C and 37°C, the exocyst components were coprecipitated with Sec5-GFP. Our result suggests that the assembly of the exocyst complex is largely unaffected in the *gic1∆gic2∆sec3∆N* triple mutant. The representative results from three independent experiments are shown.

**Additional file 3: Figure S3.** The *cdc42* mutants were not able to bind to Sec3N in vitro with either GDP or GTP in the binding buffer. GST fusion proteins containing (a.a.71–241) of Sec3 was purified and conjugated to glutathione Sepharose. Cdc42, *cdc42-301, cdc42-302, cdc42-303, cdc42-304 and cdc42-305* were expressed as a Hisx6 fusion and purified from bacteria. The in vitro binding assay was performed using GST-Sec3 and Hisx6-Cdc42 in the presence of GTPγS or GDP. The Hisx6-Cdc42 fusion protein bound to the GST-Sec3N Sepharose was detected by Western blotting with anti-Hisx6 antibody (bottom). Equal amounts of Sec3 fusion proteins on beads were used in the binding assay (middle; Ponceau S staining). Equal amounts of cdc42 wild type and mutants were used (top, 1% input, Western blotting with anti-Hisx6 antibody). The representative results from three different experiments are shown. The representative results from three independent experiments are shown.

**Additional file 4: Table S1.** Yeast strains and genotypes. **Table S2.** Plasmids used in this study.
