# Supplementary material for: The Cdc42 effectors Gic1 and Gic2 regulate polarized post-Golgi secretion
Source: Cell Biosci. 2019 Apr 4;9:33. doi: 10.1186/s13578-019-0295-x (PMC6449940; doi:10.1186/s13578-019-0295-x)
Supplement: Supplementary file 4 — Additional file 4: Table S1. Yeast strains and genotypes. Table S2. Plasmids used in this study. [file 13578_2019_295_MOESM4_ESM.docx]

**ADDITIONAL MATERIALS:**

**Table S1. Yeast strains and genotypes**

| **Strain** | **Genotype** | **Source** |
| --- | --- | --- |
| GY3652 | *MATa trp1 leu2 ura3 his3 lys2 cdc42Δ::HIS3, (pRS316-GAL1-CDC42, CEN URA3)* | This study |
| GY3668 (*cdc42-301*) | *MATa trp1 leu2 ura3 his3 lys2 cdc42Δ::HIS3, (pRS314-cdc42 V36A CEN TRP1)* | This study |
| GY3669 (*cdc42-302*) | *MATa trp1 leu2 ura3 his3 lys2 cdc42Δ::HIS3, (pRS314-cdc42 V36A F37A CEN TRP1)* | This study |
| GY3670 (*cdc42-303*) | *MATa trp1 leu2 ura3 his3 lys2 cdc42Δ::HIS3, (pRS314-cdc42 Q61A CEN TRP1)* | This study |
| GY3671 (*cdc42-304*) | *MATa trp1 leu2 ura3 his3 lys2 cdc42Δ::HIS3, (pRS314-cdc42 Y64A CEN TRP1)* | This study |
| GY3672 (*cdc42-305*) | *MATa trp1 leu2 ura3 his3 lys2 cdc42Δ::HIS3, (pRS314-cdc42 Y64A R66A CEN TRP1)* | This study |
| YEF1194 | *MATa trp1, leu2, ura3, his3, lys2* | Bi E. |
| YEF4523 | *Mata, his3-200, ura3-52 leu2-3,112 trp1-1 lys2-801* | Bi E. |
| YEF1550 | *Matα, gic1∆::LEU2, gic2∆::TRP1, his3-200, ura3-52, leu2-3,112 trp1-1, lys2-801* | Bi E. |
| GLY1003 | *Matα his2-∆200,ura3-52, leu2-3,112 trp1-1, lys2-801, sec3∆::KanMX6, (sec3∆N HIS CEN)* | This study |
| GY3055 | *Matα gic1∆::LEU2, gic2∆::TRP1, his2-∆200,ura3-52, leu2-3,112 trp1-1, lys2-801, sec3∆::KanMX6, (sec3∆N HIS CEN)* | This study |
| GY1831 | *Mata leu2-3, 112, ura3-52,* (*SEC8-13MYC, LEU2*, integrated) | This study |
| GY1864 | *Mata leu2-3, 112, ura3-52,* (*EXO84-12MYC, LEU2*, integrated) | This study |
| GY3457 | *Mata leu2-3, 112, ura3-52, (pRS316-ADH-GIC2-GFP, CEN, URA), (SEC8-13MYC, LEU2*, integrated) | This study |
| GY3458 | *Mata leu2-3, 112, ura3-52, (pRS316-ADH-GIC2-GFP, CEN, URA), (EXO84-12MYC, LEU2,* integrated) | This study |
| NY784 | *Mata leu2-3, 112, ura3-52, sec10-2* | Novick P |
| NY179 | *Mata leu2-3, 112, ura3-52* | Novick P |

**Table S2. Plasmids used in this study**

| **Plasmid** | **Description** | **Source** |
| --- | --- | --- |
| pG1664 | *pRS314-CDC42* | This study |
| pG1665 | *pRS314-cdc42 V36A (CEN TRP1)*(*cdc42-301*) | This study |
| pG1666 | *pRS314-cdc42 V36A F37A (CEN TRP1)(cdc42-302)* | This study |
| pG1667 | *pRS314-cdc42 Q61A (CEN TRP1)(cdc42-303)* | This study |
| pG1668 | *pRS314-cdc42 Y64A (CEN TRP1)(cdc42-304)* | This study |
| pG1669 | *pRS314-cdc42 Y64A R66A (CEN TRP1)(cdc42-305)* | This study |
| pNB881 | *pRS306-SEC5-GFP* | Novick P |
| pNB885 | *pRS306-SEC8- GFP* | Novick P |
| pNB880 | *pRS306-EXO70- GFP* | Novick P |
| pNB884 | *pRS306-EXO84-GFP* | Novick P |
| pG1445 | *GST-Sec3N (71-241 a.a.) in pGEX-4T-1* | This study |
| pG1424 | *GST-Gic2N (1-155 aa.) in pGEX4-1* | This study |
| pG1671 | *His6-Cdc42 in pET32a* | This study |
| pG1672 | *His6-cdc42-301 in pET32a* | This study |
| pG1673 | *His6-cdc42-302 in pET32a* | This study |
| pG1674 | *His6-cdc42-303 in pET32a* | This study |
| pG1675 | *His6-cdc42-304 in pET32a* | This study |
| pG1676 | *His6-cdc42-305 in pET32a* | This study |
| pG1216 | *p416TEF-SEC3* | This study |
| pG1388 | *pRS313-sec3∆N* | This study |
